# Healthcare professionals as domestic abuse survivors: workplace impact and support-seeking

**DOI:** 10.1093/occmed/kqae070

**Published:** 2024-08-21

**Authors:** Sandi Dheensa, Janine Doughty, Alison Gregory

**Affiliations:** Domestic Violence and Abuse Health Research Group, Centre for Academic Primary Care, Bristol Medical School, Population Health Sciences, University of Bristol, Bristol, BS8 2PS, UK; School of Dentistry, University of Liverpool, Liverpool, UK; Institute of Epidemiology and Health Care, University College London, London, UK; Domestic Violence and Abuse Health Research Group, Centre for Academic Primary Care, Bristol Medical School, Population Health Sciences, University of Bristol, Bristol, BS8 2PS, UK

## Abstract

**Background:**

Healthcare professionals (HCPs) are expected to identify and respond to domestic abuse (DA) among their patients. Although research suggests that a high proportion of HCPs are affected by DA, the impact of their experiences has been under-researched.

**Aims:**

To assess UK HCPs’ experiences of DA and develop a broad understanding of its impact on work and HCPs’ support needs.

**Methods:**

An online cross-sectional survey was promoted via multiple professional channels (October to December 2022). We adopted convenience sampling and analysed data descriptively.

**Results:**

Among the 192 HCP survivors who responded, all abuse subtypes—psychological, sexual, economic and physical—were common. Ninety per cent of abusers were male (ex)partners. Eighty-five per cent reported abusers directly interfered with their work and 92% reported their work and career were affected. Almost all reported physical and mental health consequences. Eighty-nine per cent reported their own experiences shaped their responses to patient survivors. On average, per year, HCP survivors reported they had 13 sick days, 5 days’ leave, 10 days’ lateness and 6 days’ early departure due to DA. Only 20% reported their workplace had a staff DA policy, and over 50% were unsure what workplace support mechanisms were available. Just over half disclosed at work; concerns that others would question their fitness to practice were common. Twenty-two per cent reported aspects of work, for example, long hours, stopped them from seeking support outside work.

**Conclusions:**

HCPs face unique barriers to DA disclosure and support-seeking and may benefit from tailored support from specialists who understand both DA and the healthcare context.

## Teaser

Domestic abuse (DA) is highly prevalent and damages health. Healthcare professionals are expected to identify DA and refer patients to support services, but scant research has explored healthcare professionals’ own experiences of DA. Our study highlights the wide-ranging harms of DA on their work and careers, including on patient care. Numerous work-related barriers hinder support-seeking both at work and from specialist services.

**Key learning points**

**What is already known about this subject:**

- DA is highly prevalent in England and Wales, leads to wide-ranging and long-lasting mental and physical health consequences, and can end in suicide or homicide, but advocacy and psychotherapeutic support can improve mental health and safety.
- Healthcare professionals are likely to receive disclosures of domestic abuse and have opportunities to refer survivors for support, so national and international policy emphasizes their role in asking and responding, but despite a high percentage of healthcare professionals experiencing domestic abuse themselves, research and policy has side-lined the work-related impact of their own experiences.
- Employers have a duty of care to consider how domestic abuse affects their employees, but UK-based research on this topic is lacking: the scant research indicates that staff domestic abuse policies in hospital trusts are uncommon, and that doctors, nurses and maternity professionals face unique barriers to seeking support.

**What this study adds:**

- We explored the experiences of a range of healthcare professionals and found that abusers directly interfered in respondents’ work (e.g. pre-work sleep deprivation, harassment), that abuse harmed day-to-day productivity and career advancement, and that abuse led to an average of 13 sick days annually.
- Most respondents were unsure whether their workplace had a staff domestic abuse policy, and over half reported they did not know what workplace support was available for domestic abuse: very few indicated the types of support that were available, and these were largely short-term practical support mechanisms.
- Despite the impact on work, few respondents sought support at or outside of work, many faced work-related barriers, and not all respondents felt believed when disclosing: colleagues and managers were the most common sources of support, and less than 10% sought support from occupational health.

**What impact this may have on practice or policy:**

- UK policy-makers and professional regulators recognize that healthcare professionals are experiencing all-time highs of stress, mental ill health, burnout and suicide ideation, leading to a declining workforce: domestic abuse contributes to these phenomena and UK policy-makers are starting to acknowledge the importance of a UK National Health Service response to affected staff.
- Our study highlights an immediate need for: wider implementation of basic support, particularly related to leave options and support following sick leave; longer-term/emotional support options; support options to be codified in policy; campaigns to make healthcare professional survivors aware of available options and policies; and the exploration of tailored support interventions.
- Underscoring earlier calls for domestic abuse to be seen as an occupational health issue, domestic abuse training for, and improved support from, occupational health staff, well-being services, and employee assistance programme staff could lead to benefit for healthcare professional survivors, their patients and their workforces.

## Introduction

In England and Wales, 20.5% of adults (27% women, 13.9% men) have lifetime domestic abuse (DA) experience, and 4.4% of adults (5.7% women, 3.2% men) have past-year experience [1]. File 1 (available as Supplementary data at *Occupational Medicine* online) contains contextual information about DA. DA survivors face increased depression, anxiety, post-traumatic stress disorder [2,3], suicide and suicide attempt risks [4]. Physical consequences are wide-ranging and long-lasting, including gynaecological, cardiac, and gastrointestinal problems and chronic pain [5]. Three to four domestic homicides happen in England and Wales weekly [1].

Specialist advocacy and psychotherapeutic support improve survivors’ physical and mental health and safety [6]. As trusted professionals, healthcare professionals (HCPs) are especially likely to receive DA disclosures and have opportunities to refer survivors for this specialist support [7]. Primary and community care HCPs, in particular, have multiple opportunities to engage with both perpetrators and survivors [8]. Thus, for 20+ years, UK [9] and international [10] policy has emphasized that HCPs should enquire about DA. Healthcare-based training interventions have significantly increased enquiry, identification and referral rates [11–14].

Against this backdrop, HCPs’ personal DA experiences have been side-lined. Yet, a global meta-analysis indicates 42% of female HCPs have experienced DA [15]. UK research with nurses has shown a higher prevalence than in the general population [16] and a 10-year femicide census identified HCPs as one of the commonest ‘victim occupations’ [17]. DA disproportionately affects women [1], and the UK National Health Service (NHS) is a highly feminized workforce: many NHS staff are likely affected. Recent studies highlight that HCP-perpetrated sexual misconduct towards colleagues is common but links to DA are unreported [18,19].

The UK Domestic Abuse Act 2021 statutory guidance [9] highlights a duty of care on employers to consider how DA affects employees. NHS England has a DA policy for its own staff, and NHS Employers [20] has a template policy for NHS workplaces to adopt, recognizing that ‘abusive and violent behaviour … can frequently cross over into the workplace … [and] work can be a lifeline to independence and survival’. Work can bring social, financial and other support, and a sense of agency, strength and positive self-identity, but HCP survivors unsupported at work can feel further traumatized [21]. NHS Employers’ template policy targets managers who support employees. It recommends support mechanisms that should be available, predominantly practical measures to address acute risk situations. However, 2 years after the template’s original publication, 32% of secondary care trusts and health boards had not implemented a policy, and just 1% of policies implemented listed all the support mechanisms recommended [22].

HCPs can face unique barriers to disclosure and support-seeking [15], which increases risks of further harm and death [6]. Three qualitative studies (only one peer-reviewed [23]) of UK doctors, nurses and maternity HCP survivors of DA highlight fears of fitness to practice or regulator reviews as barriers. This research also shows that survivors can ‘shut down’ when faced with patients experiencing DA [23–25]. With just three small-scale studies, little UK-based research has explored HCP survivors’ experiences and work impacts. Therefore, we aimed to assess HCPs’ DA experiences, particularly across primary and community specialities, to develop a broad understanding of work impacts and support needs.

## Methods

The study team comprised primary and community healthcare clinical academics and HCP survivors. We developed an anonymous, confidential, cross-sectional survey to explore the experiences of HCP survivors and staff who support colleagues (e.g. line managers). Respondents could complete it as a survivor, supporter or both. This article provides an overview of HCP survivor data. The survey targeted survivors who had already framed their experiences as DA so we could explore their support-seeking experiences. Thus, we anticipated most respondents to have past, rather than current, experience. No criteria excluded participation except not working in the UK.

Survey items for HCP survivors captured demographic details, types of DA experienced, impact on work and health, and support-seeking experiences. One item asked whether experiences were current or ‘within last 12 months/1–5/6–10/11+ years ago’. We drew on the validated Abusive Workplace Disruptions Assessment tool [26], earlier research [15] and team suggestions to develop survey items regarding abusive behaviours that directly interfered with work. Survey items included questions about support measures, including measures that the NHS Employers [20] template policy and the NHS England in-house staff DA policy list, and measures that HCP survivors within the study team highlighted as important. Most questions were multiple choice: respondents could tick all those that applied. The study team fed back on survey drafts that SD and AG developed. Following revisions, we migrated the survey online. Study data were collected and managed using REDCap electronic data capture tools hosted at University of Bristol. Fifteen people, including four HCP survivors, piloted it and gave more detailed feedback. Final revisions were made.

We launched the survey on 29 September 2022 following approval from University of Bristol’s Faculty of Health Sciences Research Ethics Committee (1139) (see File 2 for survey, available as Supplementary data at *Occupational Medicine* online). Although it was online, information pages detailed alternative access options (no one took these up). Information pages encouraged respondents to skip questions, take breaks, or stop if needed, and detailed the data withdrawal process. We used convenience sampling. To advertise the survey, we circulated the web link to primary care network directors, who cascaded the information to general practices in their areas, safeguarding and communications leads at English community hospitals, and via social media, tagging relevant accounts with large followings (e.g. Pulse Today, Royal Pharmaceutical Society, College of General Dentistry). Advertisements used the terms ‘domestic abuse/coercive control’ to capture HCP survivors who used one or both terms to define their experience. We, moreover, raised study awareness during dentistry staff training, a regional pharmacist networking event and general practice journal discussion article [27]. We primarily targeted primary and community HCPs as these professionals often engage with survivors in their work, and from England, to make NHS England-specific recommendations, but we did not exclude respondents from other areas. We also included responses from HCPs who were not working in healthcare at the time of DA, as abuse has long-lasting health sequelae. Given the wide advertisement for the survey, determining the numbers our survey reached and thus a response rate was not possible. The survey closed on 9 December 2022 coinciding with the end of ‘16 days of activism against gender-based violence’, a global campaign. We report frequencies and means, calculated within REDCap. Denominators fluctuate, as not all respondents completed the survey, and are indicated in tables. We round percentages to whole numbers. We used a largely deductive basic content analysis [28] to code and categorize free-text answers to ‘other: please specify’ questions, which we illustrate with quotations. Forthcoming articles will present detailed free-text analysis.

## Results

One hundred and ninety-two HCPs who had experienced DA responded: 21% (*n* = 41) also had a role supporting staff who had experienced DA. Forty-eight completed the survey partially. We received no requests to withdraw data. Ninety-eight per cent of respondents were England based (with 1% [*n* = 2] Scotland based, and <1% [*n* = 1] each Wales based and Northern Ireland based). Most (96%, *n* = 132) were women, 3% (*n* = 4) were men and <1% (*n* = 1) was non-binary. Participants were heterosexual (90%, *n* = 122), bisexual or gay/lesbian (3%, *n* = 4 each), pansexual (2%, *n* = 3), or preferred not to say (2%, *n* = 2). Table 1 details areas of work, age ranges and ethnicity: 91% were White. Thirty respondents worked outside health care when they experienced DA.

**Table 1.** Respondents’ areas of work, age ranges, and ethnicities

Area of healthcare practice	*n* (%) of total sample (*n* = 190)
Community hospital or service	59 (31)
General practice	51 (27)
Dentistry*	23* (12)
Secondary care trust	18 (9)
Pharmacy	12 (6)
Commissioning or administration	6 (3)
Safeguarding/criminal justice within a healthcare trust	5 (3)
Sexual health; ambulance services; palliative/hospice care; mental health; ‘other’ with no further detail	2 (1 each)
Musculoskeletal; other nursing; screening/immunisations; substance misuse; charity; domiciliary	1 (< 1 each)
**Job role**	***n* (%) of total sample (*n* = 190)**
Nurse	34 (18)
General practitioner (GP)	24 (13)
Healthcare support worker/assistant	15 (8)
Non-clinical administrative roles: two were also counsellors	13 (7)
Pharmacist	11 (6)
Non-clinical managers; dentists	9 each (5 each)
Dental hygienist or therapist	8 (4)
Dental nurse/technician	6 (3)
Allied healthcare professional; community hospital nurse practitioner	5 each (3 each)
Staff grade/speciality doctor; general practice managers; safeguarding leads/advisors; non-clinical informatics/educator roles	4 each (2 each)
General practice nurse practitioner; health visitor; consultant (doctor); community paramedic; care coordinator	3 each (2 each)
** **Pharmacy assistant/technician; doctor-in-training; other nurse; mental health worker/practitioner; physicians’ associate; social worker; anonymized roles	2 each (1 each)
** **Practice nurse; midwife; psychologist	1 each (< 1 each)
**Age**	***n* (%) of total sample (*n* = 137)**
** **36–45; 46–55	42 each (31 each)
** **56–65	26 (19)
** **26–35	24 (18)
** **18–25	2 (1)
** **66+	1 (< 1)
**Ethnicity**	***n* (%) of total sample (*n* = 137)**
** **White: English/Welsh/Scottish/Northern Irish/British	120 (88)
** **Other White	4 (3)
** **Prefer not to say, Indian	3 each (2 each)
** **Black African, Bangladeshi	2 each (1 each)
** **Black Caribbean, other mixed, other Black	1 each (< 1 each)

^*^ *n* = 9 in a fully private practice: otherwise, respondents worked for the NHS or in an NHS England commissioned service.

Ninety per cent (*n* = 171) of respondents’ abusers were male (ex)partners. Twenty-one per cent (*n* = 40) had a male partner and one or more other abusers (mainly parent(s)) totalling 251 abusers, and 11% (27/251) of abusers worked in health care. All abuse subtypes—psychological, sexual, economic and physical—were common. Over 1 in 10 (37%, *n* = 70) respondents reported currently experiencing DA. The remainder reported experiencing DA within the last 12 months (4%, *n* = 8), or 1–5 (16%, *n* = 30), 6–10 (19%, *n* = 37) or 11 or more (24%, *n* = 46) years ago.

Of respondents who worked in health care at the time of the abuse, 85% (*n* = 125) reported that the abuser directly interfered in their work in one or more ways, including pre-work sleep deprivation, harassment at work and accusations of infidelity with colleagues or patients. Table 2 contains more details.

**Table 2.** DA behaviours that directly interfered with HCPs’ work and percentage and number that experienced the different behaviours

Behaviour	*n* (%) of subsample working in healthcare at time of abuse (*n* = 147)
Did not let me sleep, or sleep well, before I went to work	76 (52)
Emailed, called, or messaged me many times a day while I was at work [harassment]	66 (45)
Accused me of having romantic relationships with, or sleeping with, colleagues or patients	63 (43)
Prevented me from accessing the opportunities or education I needed for my career	53 (36)
Made it difficult to leave my children when I needed to work	47 (32)
Did something else that interfered with my work [free text]	43 (29)
Did something to affect my means of getting to work	36 (24)
Followed me when I went to work or hung around outside where I was working [stalking]	28 (19)
Interacted with my colleagues in an inappropriate or abusive way	23 (16)
Came to work and interacted with patients in an inappropriate or abusive way	1 (<1)

Free-text comments described economic abuse: being coerced into particular roles or working hours: for example, into working more to earn more, even when ill, or into working less to do childcare and domestic labour. Free-text comments additionally described abusers making, or threatening to make, malicious allegations to colleagues and professional regulators, and creating conflict or bruising to upset or ‘embarrass’ respondents pre-work. Nine said in free text that they lost or resigned from their jobs due to abuse and its consequences.

Of the total sample, 97% (*n* = 166) reported physical and/or mental health harms and 92% (*n* = 158) indirect effects on work, detailed in Table 3. These were commonly impaired performance related to concentration, confidence in abilities, memory and pace. Respondents were also triggered, and felt unsafe, at work. Results were similar for the subsample working in health care at the time of the abuse. Free-text comments described how the abusers’ constant belittling and subtle psychological abuse harmed respondents’ confidence around their responsibilities at work.

**Table 3.** Indirect impact on work and percentage and number that experienced these effects

Impact on work	*n* (%) of total sample (*n* = 171)	*n* (%) of subsample working in healthcare at time of abuse (*n* = 147)
I could not concentrate at work	123 (72)	108 (73)
I did not feel confident about my ability to do my job	106 (62)	93 (63)
I had difficulty remembering what tasks to do at work	78 (46)	71 (48)
I had a noticeably slower pace when completing tasks	72 (42)	66 (45)
I was triggered at work	62 (36)	58 (39)
I did not take promotions or opportunities for advancement	61 (36)	55 (37)
I felt unsafe at work	39 (23)	35 (24)
It affected me in another way [Free text]	32 (19)	31 (21)

Annually, due to abuse, based on respondents’ self-reports on the survey, they took a mean of 13 sick days (95% CI 7.9–17.7, range of 0–183, *n* = 150) and 5 annual leave days (95% CI 3.6–6.9, range of 0–75, *n* = 147), and had 10 days’ lateness (95% CI 5.8–14.3, range of 0–235, *n* = 147), and 6 days’ early departure (95% CI 3.5–8, range of 0–100, *n*=145). Free-text comments highlighted that direct physical injury, longer-term physical and mental health problems, sleep deprivation, and childcare resulted in time off, and accusations of infidelity and ‘guilt-tripping’ around childcare and domestic labour pressured respondents to leave work early.

Eighty-four per cent (*n* = 123) of respondents had seen at least 1 patient experiencing DA and 39% (*n* = 57) had seen 11 or more within the past 5 years. Of these respondents, most (89%, 110/123) reported their identification and response to patients were affected, mostly improved recognition of DA, and a more empathic and knowledgeable response. However, free-text comments illustrated that negative reactions often accompanied these positive outcomes: being triggered, re-traumatized, overwhelmed and drained. Others reported *only* negative reactions, including acute trauma responses such as freezing, shaking and nausea. Seventy-two per cent (*n* = 107) received DA training during or after their own experience: again, many were triggered (‘It reignited … anger grief fear and rage’); others realized, for the first time, what was happening to them (‘[It] first sowed the seed … that I may be experiencing [DA]’).

When asked whether their workplaces had current staff DA policies, most respondents were unsure (67%, *n* = 78), 21% (*n* = 25, including *n* = 6 general practice, *n* = 2 dentistry, *n* = 1 pharmacy, *n* = 1 community hospital/service) said yes, and 12% (*n* = 14, including *n* = 6 general practice, *n* = 2 dentistry, *n* = 1 pharmacy, *n* = 1 community hospital/service) said no.

Regarding workplace support mechanisms available, 51% (*n* = 83) were unsure what was available. Others indicated that specific types of support were available: Table 4 contains more details and shows that for each support mechanism, only small percentages said it was available. ‘Confidentiality assurances’ aside, the commonest mechanisms were ‘changes to working times/days/patterns’, occupational health (OH) referrals and special leave (of the total sample, 17%, 14%, and 12% said these were available respectively).

**Table 4.** Workplace support mechanisms, policy of origin and percentage and number who reported these were available

Workplace support mechanism			
	*n* (%) who indicated it was available		Recommended in …?
	Total sample (*n* = 163)	Current abuse (*n* = 59)	• NHS Employers template • NHS England policy • Both • Neither
**Working hours** **and duties**			
** **Changes to working times, days, or patterns	27 (17)	13 (22)	Both
** **Changes to specific duties (e.g. to avoid contact with the abuser(s))	9 (6)	5 (8)	NHS England
** **Not being asked to do the usual return-to-work process after sick leave	8 (5)	3 (5)	Both
** **The option for redeployment or relocation	4 (2)	1 (2)	Both
**Le** **ave**			
** **Special leave provisions including unpaid leave	20 (12)	7 (12)	Both
** **Permission to attend appointments related to DA during work hours	14 (9)	7 (12)	Neither
** **Permission to use private spaces at work to hold relevant appointments	7 (4)	5 (8)	Neither
**Safety** **planning**			
** **Measures to ensure safety at work (e.g. screening calls, security alerted)	15 (9)	8 (14)	Both
** **Permission to use work phones and computers to access information and support	13 (8)	6 (10)	Neither
** **Review of personal information held by the workplace, e.g. address	10 (6)	6 (10)	NHS England
** **Measures to ensure safety while travelling to and from work	8 (5)	5 (8)	Both
** **Training for security and reception staff about what to do if the abuser(s) show up	6 (4)	4 (7)	Neither
** **Option to stay at work for safety (e.g. to stay late or to sleep at work)	5 (3)	2 (3)	Neither
**Referrals and** **signposting**			
** **Referral to OH	23 (14)	13 (22)	Both
** **Support from qualified professionals (e.g. staff counsellors, therapists)	17 (10)	10 (17)	Neither
** **Referral to an employee assistance programme	12 (7)	6 (10)	Both
** **Signposting to an in-house independent domestic violence advisor or advocate	11 (7)	7 (12)	NHS England
**Pa** **y**			
** **Referral to a credit union or financial advisory service	2 (1)	0 (NA)	NHS England
** **Changes to pay arrangements	2 (1)	2 (3)	NHS Employers template
**Confiden** **tiality**			
** **Reassurance that disclosure would be kept confidential	44 (27)	19 (32)	NHS Employers template

Looking at Table 4’s ‘current DA’ subsample, for most support mechanisms, a slightly higher percentage said the mechanism was available compared with the total sample, suggesting that mechanisms were becoming more common, but percentages were still relatively small (e.g. 22% said changes to working times/days/patterns). Of the ‘past DA’ subsample, 2% (*n* = 2) reported workplace counselling was implemented since their own experience.

Ten per cent (*n* = 14) of the total sample reported a current DA worker who provides staff support, although not formally part of their (patient-facing) role in a third of cases.

Ninety per cent (137/152) faced barriers to disclosing and/or seeking support from someone at work, organized into themes (Table 5) relating to perceptions of professionalism, fear (of colleagues’ reactions, impact on career; abuser retaliation) and HCP identity (‘DA should not happen to HCPs’).

**Table 5.** Barriers to disclosure at work and percentage and number that experienced the different barriers

Barrier	Theme	*n* (%) of total sample (*n* = 152)
I felt that I should keep my work and home life separate	Perceptions of professionalism	99 (65)
I thought that people at work would judge, blame, or think less of me	Fear of colleagues’ reactions	91 (60)
I didn’t think that it was anyone’s role to support with DA	Perceptions of professionalism	64 (42)
I felt that DA should not happen to someone in my role, or to a HCP	HCP identity	58 (38)
I worried that it would affect my professional registration or make people question my fitness to practice	Fear of impact on career	57 (38)
I didn’t think people at work would believe me	Fear of colleagues’ reactions	55 (36)
I worried that it would affect my career direction or progression	Fear of impact on career	55 (36)
I was scared that the abusive person/people would find out	Fear of abuser retaliation	40 (26)
I experienced other barriers [from free text: commonly worries about children being taken into care, not recognizing my own experience as DA, non-supportive work environment, fear of consequences to the abuser(s)]		22 (14)

**Figure 1.**
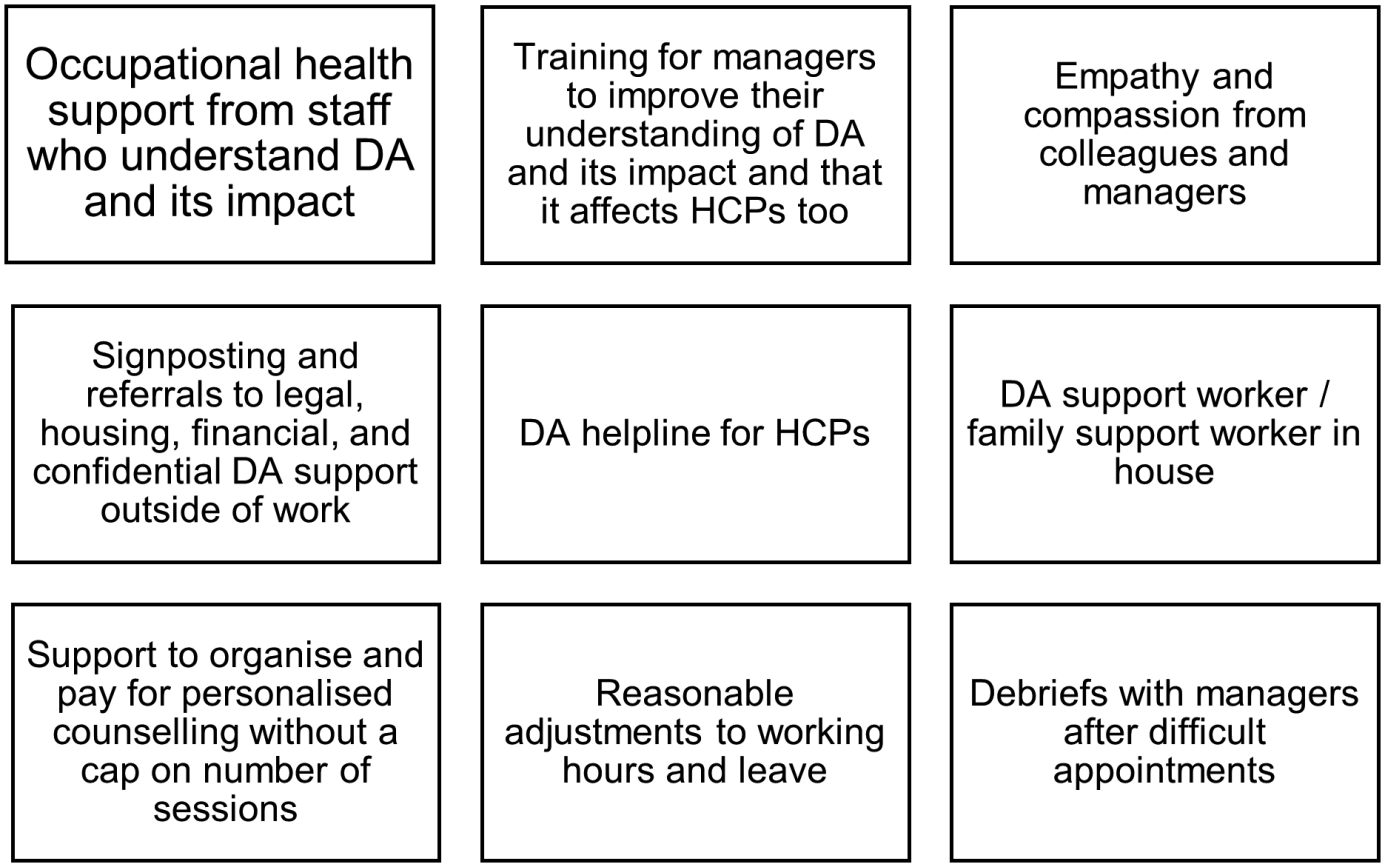
Support that was not available but would have been helpful: in addition to those listed in Table 4.

Some respondents eventually disclosed and/or sought support at work despite these barriers: 54% (83/154) reported doing so; 44% (67/154) did not. Those who did mostly sought support from colleagues (38%, *n* = 58/154) and managers/supervisors (37%, *n* = 57/154). Just 7% (*n* = 11), 6% (*n* = 9) and 3% (*n* = 5) sought OH, staff well-being and employee assistance programme support, respectively. Four free-text comments were about OH: three GP respondents would have found OH input helpful, but one community nurse felt ‘persecuted’ by her OH doctor. Linking to this finding, upon disclosure of abuse, 22% (*n* = 17) were unsure whether the person believed them, and 5% (*n* = 4) felt disbelieved. Free-text comments indicated that simply being believed and listened to were helpful aspects of support following disclosure. Of the 44% (*n* = 67) who did not disclose and/or seek support from someone at work, most did not know what support was available, but 21% (14/67) indicated that although support was available, they chose not to take it up. Types of support that were not available but were described as potentially helpful are summarized in Figure 1.

Elements of work affected the ability to seek support from outside of work for 22% of respondents (*n* = 33), including working hours (16%, *n* = 24), worries about seeing patients at specialist services (8%, *n* = 12) and ineligibility for certain support types (3%, *n* = 3). Free-text comments highlighted respondents wished not to be ‘clients’ of services with which they had professional relationships and felt pressured to retain a ‘highly functional’ HCP image.

## Discussion

Current DA was reported by over 1 in 10 respondents, was perpetrated mainly by male partners and 11% of abusers were healthcare workers. Abuse affected work and health, and led to absence and lateness. Abuse rippled out to others in the workplace (accusations of infidelity with patients/colleagues, abusive interactions with colleagues, malicious allegations to colleagues and regulators). An adverse impact on patient care was reported. Personal experience improved identification and response to patient survivors, but, along with DA training, led to being triggered at work. Respondents, moreover, felt unsafe at work. Policies and support options were lacking and numerous barriers to disclosure and support-seeking related to respondents’ roles as HCPs.

Our study is the first to describe the impact of DA on a range of UK HCPs. A greater number of survivors currently experiencing abuse may have responded if study advertisements avoided the terms ‘DA/coercive control’, as recognising and naming DA can take time. We could not determine a response rate. Experiences might have been many years ago: nevertheless, these were relevant as consequences can be long-lasting. Not all respondents completed the survey, but offering a ‘stop partway’ option was important to protect participant well-being. Few men and people of minoritized ethnicities, sexualities and genders, and from pharmacy and sexual health, participated, limiting generalizability and ability to capture intersecting harms (e.g. institutional racism). Our sample size outweighed that of a comparable UK survey for maternity HCPs [24] but was too small for meaningful subgroup analysis. The small sample size also limits the breadth of experience captured.

Our research complements earlier research. Regarding sleep deprivation as an abusive form of work interference, a Finnish study with HCP survivors showed that DA also *indirectly* affects sleep, and sleep quality mediates the relationship between DA and depression [29]. Thus sleep deprivation intensifies the impact of other abuse types, and is linked to burnout and physical and mental health problems for HCPs [30, 31]. A UK Trade Unions Congress (TUC) survey also highlighted abusers’ interference with work (e.g. stalking), barriers to disclosure in the workplace, and impact on lateness, leave and performance, as important issues [32]. Estimated annual DA-related costs to the England and Wales economy are £14m from lost output and £2m from treating healthcare sequelae: HCPs’ DA experience is thus an expensive problem for the NHS [33].

Echoing an earlier secondary care-based investigation [22], we found patchy implementation of staff DA policies and that implemented policies rarely cited the support mechanisms recommended by NHS Employers [20]. This finding is concerning given that abuse extended to the workplace. Less than 10% of respondents indicated current safety measures for work-related travel, putting community HCP survivors at particular risk, for example. Adjustments to the post-sickness return-to-work process were infrequently available, despite sick leave being one of the few recourses HCP survivors had. Longer-term and/or emotional support (e.g. therapy, counselling) was also infrequently accessed, even though responding to and attending training about DA was a common part of HCP survivors’ jobs. Financial support mechanisms were almost non-existent despite HCP survivors experiencing economic abuse and the economic consequences of time off.

Likely related to the patchy implementation of policy and support, less than half of respondents disclosed and sought support at work. Commonly, they spoke to managers, who may lack DA training, and colleagues, who may additionally lack the power to put support mechanisms in place. Support may have thus been inadequate or ineffective at enhancing safety and protecting health. The TUC moreover points out that unaware or unsympathetic managers may discipline or dismiss survivors, and that losing an ‘independent source of income is a disastrous outcome’(p.6) [32].

Our results support existing studies with UK HCP survivors [23–25], which identified barriers to disclosure and support-seeking at work, including unclear available support, ‘professionalism’ and fear. Our results show that the healthcare role, moreover, hindered support-seeking from sources outside work. Donovan and colleagues [23] similarly found that the HCPs and social workers caring for doctor survivors missed cues that these doctors were experiencing abuse and thus missed opportunities to refer them for support. Participants felt that they missed these because doctor survivors are not stereotypical victims. Other HCPs and social workers threatened to report doctor survivors to their regulator or employer [23]. The current provision of DA support, therefore, likely underserves HCP survivors.

Recent Australian research [34] has called for strengthened support and advocacy specifically for HCP survivors. A precedent for tailored support exists in England: NHS Practitioner Health provides effective mental health and addiction care specifically for HCPs [35, 36]. An OH-based trauma therapy for emergency service professionals, including those with personal or secondary DA experience has also shown promise [37].

UK policy-makers and professional regulators have recognized that HCPs are experiencing all-time highs of stress [38], mental ill health, burnout [39] and suicide ideation [40], contributing to a declining workforce [41,42]. DA contributes to these phenomena, and UK policy-makers are starting to acknowledge the importance of an NHS response to affected staff. The Women’s Health Strategy [43] specifically commits NHS England to ensure that employers, and the NHS more broadly, support survivors. NHS England has appointed a DA lead whose remit includes developing internal policies and support options. Our study contributes to these policy discussions, by highlighting what is needed immediately: wider implementation of basic safety support (as recommended by NHS Employers); support related to leave options; longer-term/emotional support options (either in-house, or signposted to); support options to be codified in policy; campaigns to make HCP survivors aware of available options and policies; and tailored support interventions delivered by specialists who understand DA and the healthcare role. These changes would convey a clear message that DA *does* happen to HCPs and that managers and other NHS staff groups, including OH and well-being services, have a role in supporting affected employees. In turn, these messages may help to dispel barriers to disclosure related to perceptions of professionalism and fears of colleagues’ reactions. Underscoring an earlier call for DA to be seen as an OH issue [44], our study also highlights that staff groups working for OH, well-being services, and employee assistance programmes, need basic training about DA, its work-related impact, and how to respond without judgement, disbelief, or victim-blaming. Training should educate these staff groups on how to support employees in safely maintaining employment if they wish to do so [45]. Their support could benefit survivors, their patients and workforces more broadly. Other barriers to disclosure require attention: specifically, professional regulators need to give clear guidance about DA and fitness to practice.

Further research should explore acceptable and effective interventions for HCP survivors. Improved support is urgently needed: DA affects HCP survivors’ work and health, which has a wider impact including on patient care, but HCPs face unique barriers to seeking the support that is essential for safety and well-being.

## Supplementary Material

kqae070_suppl_Supplementary_File_1

kqae070_suppl_Supplementary_File_2
