# Correction to: Healthcare professionals as domestic abuse survivors: workplace impact and support-seeking

**DOI:** 10.1093/occmed/kqae106

**Published:** 2024-12-10

This is a correction to: Sandi Dheensa, Janine Doughty, Alison Gregory, Healthcare professionals as domestic abuse survivors: workplace impact and support-seeking, *Occupational Medicine*, Volume 74, Issue 7, October 2024, Pages 514–522, https://doi.org/10.1093/occmed/kqae070

The following changes have been made to the originally published manuscript.

In the Introduction’s fifth paragraph, the second sentence had an erroneous reference and should read: “Three qualitative studies (only one peer-reviewed [23]) of UK doctors, […]” instead of: “Three qualitative studies (only one peer-reviewed [21]) of UK doctors, […]”.

The end of the caption for Fig. 1 is emended to read: “[…] in addition to those listed in Table 4” instead of: “[…] in addition to those listed in Table 5”.

