# Supplementary material for: Healthcare professionals as domestic abuse survivors: workplace impact and support-seeking
Source: Occup Med (Lond). 2024 Aug 21;74(7):514–22. doi: 10.1093/occmed/kqae070 (PMC11444377; doi:10.1093/occmed/kqae070)
Supplement: kqae070_suppl_Supplementary_File_1 [file kqae070_suppl_supplementary_file_1.doc]

**Supplementary file 1: Definitions and contextual information about DA.**

Background DA is a violation of basic human rights and a form of gender-based violence.

To whom the term applies: The term applies if the abuser and survivor are both aged 16 or over, and the two parties are current or ex- partners, have had a parental relationship with the same child (under 18), or are family members or in-laws.

Subtypes: It includes physical and sexual violence, and economic, psychological, and emotional abuse, enacted through intimidation, manipulation, humiliation, harassment, stalking, and threats—both to the survivor and their loved ones (e.g., children, pets, and other family members). All types of DA help the abuser to coercively control the survivor.

Incident vs pattern of behaviour: DA most often consists of a pattern of (often subtle) behaviours, rather than one-off incidents of violence, which create invisible chains that trap and control the survivor.

Most at risk: Statistically, women are more likely to experience DA than men, and men are more likely to be abusive than women. Disabled people and people with long-standing illnesses are disproportionality affected[1].
