# Supplementary material for: Healthcare professionals as domestic abuse survivors: workplace impact and support-seeking
Source: Occup Med (Lond). 2024 Aug 21;74(7):514–22. doi: 10.1093/occmed/kqae070 (PMC11444377; doi:10.1093/occmed/kqae070)
Supplement: kqae070_suppl_Supplementary_File_2 [file kqae070_suppl_supplementary_file_2.pdf]

Your Role To go back to a previous page of the survey, or to go to the next page, please use the 'Previous Page' and 'Next Page' buttons at the bottom of the screen. Do not use the back button in your browser. Please use 'Save & Return Later' at the bottom of the screen if you would like to take a break.

What area (primary or community healthcare) do you work in?

◯ General Practice
◯ Dentistry
◯ Optometry
◯ Pharmacy
◯ Community hospital or service
◯ Other [Please tell us what area of healthcare you work in] ______

◯ For a dental practice
◯ For a dental hospital
◯ Other dental

◯ For an eye hospital
◯ For an independent optometrist or optician
◯ Other optometry

◯ For a hospital pharmacy
◯ For a general practice
◯ For community pharmacy
◯ Other pharmacy

◯ For a Community Health NHS Trust
◯ For a community hospital within a larger trust
◯ Other community

Do you work for the NHS or a private practice?

◯ I work in a fully NHS service
◯ NHS England commissions my service, or part of it, and I see NHS patients, but it's a private practice
◯ It's fully private and I do not see NHS patients
◯ I'm not sure
◯ Other [Please tell us more, if you want to] ______

What is your role? (Please choose the nearest option for your role, or choose 'other' and tell us about your role)

◯ General Practitioner
◯ Doctor in training
◯ Staff grade/specialty doctor
◯ Nurse
◯ Nurse practitioner
◯ Practice nurse
◯ Community paramedic
◯ Dentist
◯ Dental nurse/technician
◯ Optometrist
◯ Optician
◯ Pharmacist
◯ Pharmacy assistant/technician
◯ Healthcare support worker/assistant
◯ Other [Please tell us what your role is] ______

Please tell us how long you have been in your current role in years or months (please specify whether you mean years or months)

____

Where is the main place that you work?

◯ England
◯ Wales
◯ Scotland
◯ Northern Ireland
◯ Other [Please tell us where you work] ______

Context To go back to a previous page of the survey, or to go to the next page, please use the 'Previous Page' and 'Next Page' buttons at the bottom of the screen. Do not use the back button in your browser. Please use 'Save & Return Later' at the bottom of the screen if you would like to take a break.

These questions are about your relationships with any person who has used coercively controlling, abusive, or violent behaviours toward you.

You may have never thought about the behaviours in these terms, but may have felt that the relationship was unhealthy, harmful, or wrong in some way.

Please note: We have written most questions in the past tense, just to keep the wording simple, but we also want to hear from you if you are currently experiencing domestic abuse/coercive control. You can skip any questions you don't want to answer.

5 Since you were 16, who has used coercively controlling, abusive, and/or violent behaviours toward you? (Please tick all that apply)

- ☐ Male partner or ex-partner (including boyfriend / husband / father of your child/ren)
- ☐ Female partner or ex-partner (including girlfriend / wife / mother of your child/ren)
- ☐ Gender non-conforming partner or ex-partner (including the parent of your child/ren)
- ☐ Mother
- ☐ Father
- ☐ Gender nonconforming parent
- ☐ Son (aged 16 or more)
- ☐ Daughter (aged 16 or more)
- ☐ Gender nonconforming child (aged 16 or more)
- ☐ In-law/s [Please tell us who this person/these people are to you, e.g., my sister-in-law] ______
- ☐ Other family member/s [Please tell us who this person/these people are to you, e.g., my uncle] ______

Are you experiencing these behaviours now?

- ○ Yes
- ○ Most of my experiences are in the past but they continue to use some of these behaviours toward me now
- ○ No, I am no longer experiencing the behaviours

How long ago did you experience these behaviours?

- ○ Within the last 12 months
- ○ 1-5 years ago
- ○ 6-10 years ago
- ○ 11 or more years ago

Were you working in healthcare at the time you experienced these behaviours?

- ○ Yes
- ○ No

Was the abusive person working in healthcare at the time they were using abusive behaviours toward you?

- ○ Yes
- ○ No

Does the abusive person/people work in a healthcare role?

- ○ Yes
- ○ No

If you have ticked more than one person in the question starting 'Since you were 16' above, please could you tell us which person/people work in a healthcare role

____

Did you, or do you, work together?

◯ Yes
◯ No

Were/are they your manager or in a position of authority over you?

◯ Yes
◯ No

Please tell us more about the ways you work(ed) together.

e.g., being around each other at work, having the same colleagues and line managers

Types and Examples of Domestic Abuse/Coercive Control

To go back to a previous page of the survey, or to go to the next page, please use the 'Previous Page' and 'Next Page' buttons at the bottom of the screen. Do not use the back button in your browser.

In the questions below, we ask whether you have experienced psychological, economic, physical, or sexual violence and abuse, and whether these experiences are in the past 12 months or longer ago. We give a few examples of the different types of abuse to help you answer the questions.

We understand that the types of abuse overlap with each other. We also recognise that in most cases, these behaviours are underpinned by coercive control and that coercive control can be difficult to describe.
These questions might be difficult to answer and the examples might be difficult to read. So please remember you can skip questions and/or take a break by clicking 'Save & Return Later' at the bottom of the screen.

7.1 Have you experienced psychological abuse, including psychological aspects of coercive control?
For example, they did something like one or more of the following:

played 'mind-games' did not let you sleep, or sleep well stopped you, or tried to stop you, from having contact with friends, family, colleagues, professionals, and others used verbal aggression towards you insulted or humiliated you threatened you, or threatened to hurt or harm a person you care about or a pet intimidated you with subtle gestures, or more obviously, like driving recklessly with you in the car harassed you by phone, text, email, or social media followed you around constantly checked up on you looked through your phone, computer, or another device without permission, or monitored your activity on these devices tried to damage your reputation, i.e., what other people think of you or used any other form of psychological abuse

- ○ Yes, I have experienced psychological abuse in the past 12 months
- ○ Yes, I have experienced psychological abuse, but not in the past 12 months
- ○ No, I have not experienced psychological abuse
- ○ I am unsure if I have experienced psychological abuse

7.2 Have you experienced economic abuse, including economic aspects of coercive control?
For example, they did something like one or more of the following:
told you when and how you could spend money told you when and how you could use things like a mobile phone or car made you justify what you spent prevented you from working, or made it difficult to work refused to contribute to household or childcare costs misused or spent money meant for other purposes (e.g., for bills, for children) stole, took, or gave away your property or money insisted all bills, credit cards, or loans were in your name, or in their name built up debt in your name or used any other form of economic (including financial) abuse

- ○ Yes, I have experienced economic abuse in the past 12 months
- ○ Yes, I have experienced economic abuse, but not in the past 12 months
- ○ No, I have not experienced economic abuse
- ○ I am unsure if I have experienced economic abuse

Have you experienced physical violence and/or abuse? For example, they did something like one or more of the following:
pushed, grabbed, shoved, or shook you pinched, bit, or burned you slapped, hit, or kicked you choked or strangled you used a knife, gun, or another weapon against you or used any other form of physical violence or abuse

- ◯ Yes, I have experienced physical violence and/or abuse in the past 12 months
- ◯ Yes, I have experienced physical violence and/or abuse, but not in the past 12 months
- ◯ No, I have not experienced physical violence and/or abuse
- ◯ I am unsure if I have experienced physical violence and/or abuse

Have you experienced sexual violence and/or abuse?

For example, they did something like one or more of the following:

touched you sexually without your consent pressured, manipulated, scared, or physically forced you into performing a sexual act made you engage in sexual activity with another person made you touch yourself

sexually forced you to watch or look at porn shared private or intimate images of me without my consent or used any other form of sexual violence or abuse

- ◯ Yes, I have experienced sexual violence and/or abuse in the past 12 months
- ◯ Yes, I have experienced sexual violence and/or abuse, but not in the past 12 months
- ◯ No, I have not experienced sexual violence and/or abuse
- ◯ I am unsure if I have experienced sexual violence and/or abuse

7.5 If you want to, you can use the box below to tell us about any other forms of coercively controlling, abusive, and/or violent behaviours that you have experienced.

7.6 You indicated (on the previous page) that the abusive person/people work(ed) in a healthcare role. With this in mind, did they do any of the following?

- ☐ Used their access to medicines to drug or sedate you
- ☐ Used their access to medicines to swap your medication without you knowing
- ☐ Encouraged or forced you to use medications that you did not want to
- ☐ Looked at your medical record without permission
- ☐ Found out your contact details using NHS records
- ☐ Used their connections, or their position, to discredit you among your healthcare colleagues
- ☐ Used their connections, or their position, to discredit you to your own GP, or to other healthcare professionals caring for you
- ☐ Did something else that took advantage of their healthcare role [Please tell us more, if you want to] ______

8 Did the abusive person's/people's behaviour ever make you feel any of the following?

8.1 Unsafe or scared

- ◯ Yes, in the last 12 months
- ◯ Yes, but not in the last 12 months
- ◯ No

That you had to change your behaviour, or choices, to meet their demands or to keep the peace

- ◯ Yes, in the last 12 months
- ◯ Yes, but not in the last 12 months
- ◯ No

8.3 That you had to stick to their rules, decisions, or routines

- ◯ Yes, in the last 12 months
- ◯ Yes, but not in the last 12 months
- ◯ No

8.4 That your freedom or options were limited

- ◯ Yes, in the last 12 months
- ◯ Yes, but not in the last 12 months
- ◯ No

8.5Humiliated, belittled, or undermined

◯ Yes, in the last 12 months
◯ Yes, but not in the last 12 months
◯ No

How Domestic Abuse/Coercive Control Affected Your Work To go back to a previous page of the survey, or to go to the next page, please use the 'Previous Page' and 'Next Page' buttons at the bottom of the screen. Do not use the back button in your browser. Please use 'Save & Return Later' at the bottom of the screen if you would like to take a break.

We have written most questions in the past tense just to keep the wording simple but we also want to hear from you if you are currently experiencing domestic abuse/coercive control.

9 The following statements describe ways that an abusive person/people may have interfered with your work. Please tick all that have happened to you.

- ☐ They did not let me sleep, or sleep well, before I went to work
- ☐ They did something to affect my means of getting to work
- ☐ They made it difficult to leave my children when I needed to work
- ☐ They emailed, called, or messaged me many times a day while I was at work
- ☐ They followed me when I went to work or hung around outside the place I was working
- ☐ They came to my work and interacted with patients in an inappropriate or abusive way
- ☐ They interacted with my colleagues in an inappropriate or abusive way
- ☐ They accused me of having romantic relationships with, or sleeping with, colleagues or patients
- ☐ They prevented me from accessing the opportunities or education I needed for my career
- ☐ They did something else that interfered with my work [Please tell us more, if you want to] ______

10 The following statements describe ways in which coercively controlling, abusive, and/or violent behaviours might have affected your work. Please tick all those that happened to you. Remember you can skip questions you do not want to answer.

- ☐ I could not concentrate at work
- ☐ I had difficulty remembering what tasks to do at work
- ☐ I had a noticeably slower pace when completing tasks at work
- ☐ I did not feel confident about my ability to do my job
- ☐ I felt unsafe at work
- ☐ I was triggered at work
- ☐ I did not take promotions or opportunities for advancement at work
- ☐ It affected me in another way [Please tell us more, if you want to] ______

11 Did the abusive behaviours affect your physical or mental health?

- ◯ Yes [Please tell us more, if you want to] ______
- ◯ Not sure [Please tell us more about this, if you want to]______
- ◯ No

11.2 Did these health issues affect your work? Or did work affect these health issues?

- ◯ Yes [Please tell us more, if you want to] ______
- ◯ No

12 For each year that you experienced domestic abuse/coercive control, please tell us the following - please give us a number, a rough average for each year:

12.1 How many days did you take off sick due to the person's/people's behaviours?

____
(On average each year)

12.2 How many days of annual leave did you take due to the person's/people's behaviours?

____
(On average each year)

12.3 How many days were you late to work due to the person's/people's behaviours?

____
(On average each year)

12.4 How many days did you have to leave work early due to the person's/people's behaviours?

____
(On average each year)

12.5 If you would like to tell us about other types of leave, or give more information about the above answers, you can do so here:

Support Available in Your Workplace To go back to a previous page of the survey, or to go to the next page, please use the 'Previous Page' and 'Next Page' buttons at the bottom of the screen. Do not use the back button in your browser. Please use 'Save & Return Later' at the bottom of the screen if you would like to take a break.

19 If you are currently experiencing domestic abuse/coercive control, or dealing with the impact and after-effects, which of the following are available in your workplace?
If your experience is in the past, please tell us what was available in your workplace at the time you were experiencing domestic abuse/coercive control, or while you were dealing with the impact and after-effects.
Please tick everything that you know is, or was, available: even things you were not offered, or did not take up. If you worked in multiple locations, please tell us about the place where you spent most of your time.

☐ I don't know what is, or was, available

Working hours and duties

☐ Changes to working times, days, or patterns
☐ Changes to specific duties (e.g., to avoid contact with the abusive person/people)
☐ Not being asked to do the usual return to work process after sick leave
☐ The option for redeployment or relocation

Leave

☐ Special leave provisions (e.g., using existing leave, or the option for unpaid leave)
☐ Paid leave for domestic abuse/coercive control
☐ Permission to attend your appointments related to domestic abuse/coercive control during work hours
☐ Permission to use private spaces at work to hold your appointments related to domestic abuse/coercive control

Safety planning

☐ Measures to ensure your safety at work (e.g., blocking emails, screening phone calls, reception and security being alerted that the abusive person/people might come to your workplace)
☐ Measures to ensure your safety while travelling to and from work
☐ Review of the personal information that the workplace holds on you, e.g address
☐ The option to stay at work for safety (e.g., to stay late or to sleep at work)
☐ Permission to use work phones and computers to look up information and access support
☐ Training for security and reception staff about how to handle situations where an abusive person turns up at a workplace

Referrals and signposting

☐ Signposting to an in-house Independent Domestic Violence Advisor (IDVA) or domestic abuse advocate
☐ Referral to an Employee Assistance Programme
☐ Referral to Occupational Health
☐ Support from qualified professionals (e.g., staff counsellors or therapists)

Pay

☐ Referral to a credit union or financial advisory service
☐ Changes to pay arrangements

Confidentiality

☐ Reassurance that my disclosure would be kept confidential

19.1 If you are currently experiencing domestic abuse/coercive control or are dealing with the impact and after-effects, please tell us about anything else that is available in the workplace, if you want to.
If your experience is in the past, please tell us about anything else that was available at the time you were experiencing domestic abuse/coercive control, or dealing with the impact and after-effects, if you want to.

21 Has any other support been introduced in your workplace since your experience?

◯ Yes [Please tell us what has been introduced] ______
◯ No
◯ I'm not sure, because I now work somewhere else
◯ I don't know

Your Experiences of Seeking Support in the Workplace To go back to a previous page of the survey, or to go to the next page, please use the 'Previous Page' and 'Next Page' buttons at the bottom of the screen. Do not use the back button in your browser. Please use 'Save & Return Later' at the bottom of the screen if you would like to take a break.

We have written most questions in the past tense just to keep the wording simple but we also want to hear from you if you are currently experiencing domestic abuse/coercive control.

13 From what you can remember, did you talk to, or seek support from, any of the following people or teams at work about your experiences of coercively controlling, abusive, and/or violent behaviours? (Please tick all that apply)

☐ Your manager or supervisor
☐ Colleagues
☐ Occupational health (OH)
☐ Human resources (HR)
☐ Employee Assistance Programme
☐ NHS staff wellbeing service
☐ Workplace counsellor or therapist
☐ Domestic abuse worker in the workplace
☐ Staff involved in any disciplinary processes [Please tell us which staff were involved, if you want to] ______
☐ Security
☐ Other people or teams [Please tell us who you spoke to, if you want to] ______

◯ I did not seek support from anyone at work
◯ I can't remember if I sought support from anyone at work

14We will now ask you a little more about your experience of seeking support.

If you sought support from more than one person or team, please tell us which person or team you are describing in your answers.
Please remember you can skip questions and/or take breaks.

14.1.W1hat events led you to talk to, or seek support from, a person or team at work?

14.1.D2id you ask for any specific help or support from them?

◯ Yes [Please tell us what help or support you asked for, if you want to] ______
◯ No

14.1.D3id you feel that the person or people believed you?

◯ Yes
◯ No
◯ Unsure

14.1.W4hat did the person or people do? (e.g., Did they offer you practical or emotional support? Did they guarantee confidentiality? Did they put any security measures in place?)

14.1.W5hat did you think of their response? By response, we mean their reaction, the way they treated you, and what they said and did

20What, if anything, was helpful for you?

Was there anything that you weren't offered, or anything that wasn't available in your workplace, that you think would have been helpful?

15 Sometimes people experience barriers or challenges to seeking help or support in the workplace. Did you experience any of the following barriers or challenges? (Please tick all that apply)

☐ I was scared that the abusive person/people would find out
☐ I didn't think that it was anyone's role to support with domestic abuse/coercive control
☐ I thought that people at work would judge, blame, or think less of me
☐ I didn't think people at work would believe me
☐ I worried that it would affect my professional registration or make people question my fitness to practice
☐ I worried that it would affect my career direction or progression
☐ I felt that domestic abuse/coercive control should not happen to someone in my role, or to a healthcare professional
☐ I felt that I should keep my work and home life separate
☐ I experienced other barriers [Please tell us more, if you want to] ______

Seeking Support from Elsewhere To go back to a previous page of the survey, or to go to the next page, please use the 'Previous Page' and 'Next Page' buttons at the bottom of the screen. Do not use the back button in your browser. Please use 'Save & Return Later' at the bottom of the screen if you would like to take a break.

22Did you talk to, or seek help from, any of the following people outside of your workplace? (Please tick all that apply)

- ☐ Friends
- ☐ Family
- ☐ Neighbours
- ☐ Police
- ☐ Religious leader
- ☐ Victim Support
- ☐ NHS therapist or counsellor
- ☐ Your own GP
- ☐ Your own healthcare professional (other than your GP)
- ☐ Solicitor or other legal professional
- ☐ Citizen's Advice Bureau
- ☐ Local or national specialist domestic abuse services
- ☐ Housing association or housing support service
- ☐ Private therapist or counsellor
- ☐ Other people, professionals, or organisations [Please tell us which people or organisations, if you want to] ______

23Did work affect your ability to seek support from any support services or organisations?

- ◯ Yes
- ◯ No

23.1In what ways did work affect your ability to seek support? (Please tick all that apply)

- ☐ Working hours made it difficult to access support
- ☐ I worried about seeing my patients at the services
- ☐ I was ineligible for certain services because of my job [Please tell us more about this, if you want to] ______
- ☐ Work affected my ability to seek support in another way [Please tell us how your ability to seek support was affected, if you want to] ______

Positive Effects of Work To go back to a previous page of the survey, or to go to the next page, please use the 'Previous Page' and 'Next Page' buttons at the bottom of the screen. Do not use the back button in your browser. Please use 'Save & Return Later' at the bottom of the screen if you would like to take a break.

Q16 Did you experience any positive effects from being at work when you were experiencing coercively controlling, abusive, and/or violent behaviours?

- ◯ Yes
- ◯ No

16.1 Please tell us more about these positive effects from being at work, if you want to

Seeing Patients Affected by Domestic Abuse/Coercive Control To go back to a previous page of the survey, or to go to the next page, please use the 'Previous Page' and 'Next Page' buttons at the bottom of the screen. Do not use the back button in your browser. Please use 'Save & Return Later' at the bottom of the screen if you would like to take a break.

17 Roughly how many patients experiencing domestic abuse/coercive control have you encountered in the past five years, as part of your work?

- ○ 0
- ○ 1-2
- ○ 3-4
- ○ 5-6
- ○ 7-8
- ○ 9-10
- ○ 11+

17.1 Do you think that your own experiences have, in any way, affected your identification and response to patients experiencing domestic abuse/coercive control?

- ○ Yes
- ○ No
- ○ Unsure - maybe

17.2 In what ways have your own experiences affected your responses to patients? (Please tick all that apply)

- ☐ It has affected my ability to recognise abuse
- ☐ It has affected my ability to respond to abuse
- ☐ There has been an emotional impact on me
- ☐ Something else

Please tell us more about this, if you want to

Training To go back to a previous page of the survey, or to go to the next page, please use the 'Previous Page' and 'Next Page' buttons at the bottom of the screen. Do not use the back button in your browser. Please use 'Save & Return Later' at the bottom of the screen if you would like to take a break.

18 Have you had any training on responding to patients experiencing domestic abuse/coercive control as part of your work?

- ○ Yes
- ○ No

18.1 Was this before, during, or after your experience of domestic abuse/coercive control?

If you have had training more than once, please tick all that apply

- ☐ Before
- ☐ During
- ☐ After

18.2 What were your experiences of this training? What did you think of it?

____

18.3 In what ways, if any, do you think that your own experiences of domestic abuse/coercive control affected your experience of the training?

____

Workplace Policy and Personnel

To go back to a previous page of the survey, or to go to the next page, please use the 'Previous Page' and 'Next Page' buttons at the bottom of the screen. Do not use the back button in your browser. Please use 'Save & Return Later' at the bottom of the screen if you would like to take a break.

24 Does your workplace have a domestic abuse policy for staff?

- ◯ Yes
- ◯ No
- ◯ Unsure

25 Does your workplace have a specialist domestic abuse worker, or someone with a designated role to support patients who are experiencing domestic abuse?

- ◯ Yes
- ◯ No
- ◯ I don't know

25.1 What is their job title? (e.g., Independent Domestic Violence Advisor (IDVA), advocate-educator, domestic abuse nurse, safeguarding lead)

____

25.2 Does this person also support staff who are experiencing domestic abuse/coercive control?

- ◯ Yes, supporting staff is part of their role
- ◯ Yes, but I don't think supporting staff is officially part of their role
- ◯ No, they do not support staff
- ◯ Unsure

About You & Opportunity for a Follow-up Interview To go back to a previous page of the survey, or to go to the next page, please use the 'Previous Page' and 'Next Page' buttons at the bottom of the screen. Do not use the back button in your browser. This is the last page - to submit the survey, please press 'Submit' at the bottom of the screen.

This is the end of our questions about domestic abuse/coercive control. If there are other things you would like to tell us, please do so in the text box below

You can also tell us if you are interested in a follow-up interview - see the question further down this page, in blue text.

The final few questions ask for information about you.

27

We ask about this 'special category personal data' to help ensure we capture experiences from a range of people. You can skip any questions you do not want to answer.

Age

28

◯ 18-25
◯ 26-35
◯ 36-45
◯ 46-55
◯ 56-65
◯ 66+
◯ Prefer not to say

29 Sex

◯ Male
◯ Female
◯ Other [Please tell us your sex, if you want to] ______
◯ Prefer not to say

30 Does your gender identity align with the sex registered at your birth?

◯ Yes
◯ No [Please tell us your gender, if you want to] ______
◯ Not applicable
◯ Prefer not to say

Are you...

◯ Male
◯ Female
◯ Non-binary/genderqueer
◯ Intersex
◯ Another term [Please tell us the term, if you want to] ______

Are you transgender?

◯ Yes
◯ No
◯ Not sure

31What is your ethnicity?

- ◯ White: English/Welsh/Scottish/Northern Irish/British
- ◯ White Irish
- ◯ Gypsy or Irish Traveller
- ◯ Other White
- ◯ White and Black Caribbean
- ◯ White and Black African
- ◯ White and Asian
- ◯ Other Mixed
- ◯ Indian
- ◯ Pakistani
- ◯ Bangladeshi
- ◯ Chinese
- ◯ Other Asian
- ◯ Black African
- ◯ Black Caribbean
- ◯ Other Black
- ◯ Arab
- ◯ Other [Please tell us your ethnicity, if you want to] ______
- ◯ Prefer not to say

32Do you have a religion?

- ◯ No religion
- ◯ Buddhist
- ◯ Christian
- ◯ Hindu
- ◯ Jewish
- ◯ Muslim
- ◯ Sikh
- ◯ Other [Please tell us your religion, if you want to] ______

33Do you have parental responsibility for children under 18?

- ◯ Yes
- ◯ No
- ◯ Prefer not to say

34What is your sexual orientation?

- ◯ Heterosexual
- ◯ Bisexual
- ◯ Gay/Lesbian
- ◯ Pansexual
- ◯ Other [Please tell us your sexual orientation, if you want to] ______
- ◯ Prefer not to say

35Do you consider yourself to have a disability, or to be Disabled? (We use both disability and Disabled here, because different people prefer different terms)

- ◯ Yes [Please tell us more about this, if you want to] ______
- ◯ No
- ◯ Prefer not to say
